# Pathophysiology of Perinatal Asphyxia in Humans and Animal Models

**DOI:** 10.3390/biomedicines10020347

**Published:** 2022-02-01

**Authors:** Daniel Mota-Rojas, Dina Villanueva-García, Alfonso Solimano, Ramon Muns, Daniel Ibarra-Ríos, Andrea Mota-Reyes

**Affiliations:** 1 Neurophysiology, Behavior and Animal Welfare Assessment, Universidad Autónoma Metropolitana (UAM), Mexico City 04960, Mexico; 2 Division of Neonatology, National Institute of Health Hospital Infantil de México Federico Gómez, Mexico City 06720, Mexico; ibarraneonato@hotmail.com; 3 Department of Pediatrics, University of British Columbia, Vancouver, BC V6H 3V4, Canada; asolimano@cw.bc.ca; 4 Livestock Production Sciences Unit, Agri-Food and Biosciences Institute, Hillsborough BT26 6DR, UK; rmunsvila@gmail.com; 5 School of Medicine and Health Sciences, TecSalud, Instituto Tecnológico y de Estudios Superiores de Monterrey (ITESM), Monterrey 64849, Mexico; andreamreyes2020@gmail.com

**Keywords:** brain injury, hypoxic–ischemic encephalopathy, human and animal models, meconium aspiration syndrome, perinatal asphyxia

## Abstract

Perinatal asphyxia is caused by lack of oxygen delivery (hypoxia) to end organs due to an hypoxemic or ischemic insult occurring in temporal proximity to labor (peripartum) or delivery (intrapartum). Hypoxic–ischemic encephalopathy is the clinical manifestation of hypoxic injury to the brain and is usually graded as mild, moderate, or severe. The search for useful biomarkers to precisely predict the severity of lesions in perinatal asphyxia and hypoxic–ischemic encephalopathy (HIE) is a field of increasing interest. As pathophysiology is not fully comprehended, the gold standard for treatment remains an active area of research. Hypothermia has proven to be an effective neuroprotective strategy and has been implemented in clinical routine. Current studies are exploring various add-on therapies, including erythropoietin, xenon, topiramate, melatonin, and stem cells. This review aims to perform an updated integration of the pathophysiological processes after perinatal asphyxia in humans and animal models to allow us to answer some questions and provide an interim update on progress in this field.

## 1. Introduction

“Globally 2.5 million children died in the first month of life in 2018, approximately 7000 newborn deaths every day, with about one third dying on the day of birth and close to three quarters dying within the first week of life” [1]. Newborn mortality differs, depending on the country. With 27 deaths per 1000 live births in 2019, sub-Saharan Africa had the highest newborn mortality rate, followed by 24 deaths per 1000 live births in central and southern Asia. An infant born in sub-Saharan Africa or southern Asia is 10 times more likely than an infant born in a high-income country (HIC) to die in the first month of life [1].

“Preterm birth, intrapartum-related complications (birth asphyxia or lack of breathing at birth), infections and congenital disabilities cause most neonatal deaths” in 2017 [1].

Progress in newborn survival has been slow, and the reduction in stillbirths has been even slower. An accelerated scaleup of care strategies targeting the major causes of death is needed in order to meet Every Newborn targets of 10 or fewer neonatal deaths and 10 or fewer stillbirths per 1000 births in every country by 2035. The most effective strategy to decrease perinatal and neonatal deaths is through interventions delivered during labor and birth, with 41% dying due to obstetric complications, and 30% followed by the care of small and ill newborn babies [2].

The incidence of neonatal hypoxic–ischemic (HI) brain injury is higher in preterm newborns than in the term newborns. Although the risk factors of perinatal asphyxia in preterm newborns are similar to those observed in the term newborns, the immature brain of preterm newborns, particularly those born before 32 weeks gestational age, is highly vulnerable to HI injury. Two primary reasons for this have been documented: (1) preterm newborns are at higher risk of hypoperfusion during transition, especially when transition is impaired; (2) their immature brains possess reduced autoregulatory capacity [3,4,5,6].

The increased survival of extremely immature infants poses an additional challenge. Data analysis on 4274 infants born after just 22–24 weeks of gestation in three periods (2000–2003, 2004–2007, 2008–2011) at the Neonatal Research Network Centers of the National Institute of Child Health and Human Development (NICHD) in the United States produced results that indicated an overall increase in survival from 30 to 36% and a rate of survival free of neurodevelopmental impairment that rose from 16 to 20% between period 1 and period 3. The frequency of cases of cerebral palsy of moderate-to-severe degree, however, did not show a significant decrease from period 1 to periods 2 and 3 (15% in period 1 vs. 11% in periods 2 and 3) [7,8,9,10]. Although evidence of this kind suggests that improvement has occurred in the affected population, indices of death and neurodevelopmental impairment, among other undesirable outcomes, are still high and raise concern that the evident decrease in mortality rates means that more infants with neurodevelopmental problems will survive. Hence, it is important to gather data on these two outcomes—death and impairment—so that physicians and family members can make informed decisions regarding early care for these high-risk children. The goal of this article is to clarify the major issues involved in the obstetric management of these cases, as well as aspects of the pathophysiology of the birth process, while stressing that it is unacceptable to decrease blood flow to the brain of fetuses and neonates in this condition [7,8,9,10]. This review aims to perform an updated integration of the pathophysiological processes after perinatal asphyxia, as well as recent investigations in different animal models, to allow us to answer some questions.

## 2. Defining Birth Asphyxia

The term birth asphyxia was introduced by the World Health Organization (WHO) in 1997 to describe the clinical condition of a newborn who either fails to establish or sustain regular breathing at birth [11,12]. In that context, birth asphyxia, implies a condition or a state in which the newborn requires immediate assistance to establish breathing. However, this makes the term imprecise and non-diagnostic of a causal pathology, which may vary from an intrapartum-related hypoxic event to a physiologic condition, such as prematurity, perinatal sedation, congenital structural abnormality of the brain or other maternal conditions.

Asphyxia has also been defined as a condition characterized by the impairment of gas exchange that can generate distinct degrees of hypoxia, hypercarbia and acidosis according to the duration and severity of airflow interruption. Asphyxia at birth—that is, impeded perinatal gas exchange—has no exact biochemical criteria, and we lack a gold standard that could ensure reliable diagnoses. For this reason, researchers have proposed numerous clinical and biochemical markers to predict, confirm or determine the condition called intrapartum asphyxia. These include verifying the pH of the umbilical cord, calculating Apgar scores, and evaluating the presence of acidosis, as well as signs of fetal distress. This is complicated, however, because intrapartum physiology can be very dynamic. These markers have limitations and remain controversial [13]. As such, caution must be exercised in labeling a neonate with asphyxia. Unfortunately, this term is often inappropriately linked with the poor neurodevelopmental outcome commonly referred to as cerebral palsy [14,15].

## 3. Circulatory Changes during Labor and Neonatal Transition

Human fetuses develop in an hypoxicemic state—but not hypoxic—where various vital mechanisms allow them to thrive. By binding to high-affinity fetal hemoglobin, for example, oxygen easily diffuses into the fetus’ bloodstream from the mother’s circulatory system. The blood that enters the placenta in this way is returned to the fetus via the umbilical vein, most of it entering the ductus venosus. The PO_2_ level of this blood ranges from 32 to 35 mmHg, but en route to the right atrium, it meets less oxygenated blood from the inferior vena cava. In the right atrium, the blood with greater oxygenation from the umbilical vein flows into the left atrium through the foramen ovale before exiting through the left ventricle to supply two arteries, the carotid and coronary. After that, it streams into the aorta with blood from the right ventricle via the ductus arteriosus [12,16]. Thus, the fetus preferentially supplies more oxygenated blood to the brain (PO_2_ of approximately 28 mmHg) and heart. The less oxygenated blood from the inferior and superior vena cava also mixes with placental blood and then exits the right side of the heart via the pulmonary trunk. Most of this blood (~90%) then bypasses the lungs via the ductus arteriosus and enters the aorta distal to the carotid and coronary arteries. The right ventricular blood has a PO_2_ of 15 to 25 mmHg; however, distal oxygen delivery is supported by the fact that a large proportion of the cardiac output of both ventricles, pumping in parallel, is systemic in the fetus [12,16]. Then, a portion of this combined ventricular output (30% during gestational weeks 20 to 30 and 20% or less at or near term) enters the placental circulation. During labor, uterine contractions lead to intermittently decreased uterine arterial blood flow and decreased flow into the intervillous spaces. Transplacental gas exchange is also impaired intermittently, but this is generally inconsequential during normal labor. When the fetal side of the circulation is examined, uterine contractions do not seem to affect umbilical blood flow. At the time when normal birth occurs, several simultaneous circulatory changes favor the adaptation of the fetus to extrauterine conditions [12,16,17,18,19].

Because circulation switches from in-parallel to in-series, there is a need to equilibrate the outflows from the left and right ventricles (LVO, RVO), but completing this process takes several days—sometimes weeks—after birth, particularly in cases of preterm infants. This may occur due to a delay in closing of the fetal channels. When infants begin to cry right after birth, pulmonary vascular resistance decreases, and the newborns’ lungs expand rapidly. Another important factor in systemic circulation is the removal of the (low-resistance) placenta by clamping the umbilical cord [20,21]. Pulmonary blood flow increases significantly, and so does the pulmonary venous return to the left atrium, closing the foramen ovale. Right-to-left shunting at the ductus arteriosus then decreases and eventually reverses as pressure of the pulmonary artery decreases below and the systemic blood pressure increases, aiding in the reversal of the ductal shunt. Increases in PaO_2_ stimulate ductal closure. Finally, especially in some very preterm neonates, the inability of the immature myocardium to pump against the suddenly increased systemic vascular resistance (SVR) might lead to a transient decrease in systemic blood flow, which in turn could also contribute to a decrease in cerebral blood flow (CBF). Thus, the transition from intrauterine to extrauterine life involves fast, complex, and well-organized steps to guarantee neonatal survival [20,21]. When all of these changes are completed, an adult circulation pattern is established [14,22].

## 4. Pathophysiology of Birth Asphyxia

Delay in starting pulmonary ventilation at birth brings about a reduction in oxygen saturation in the blood and decreased oxygen delivery to the brain, which depends on aerobic metabolism to sustain the mitochondrial respiratory chain and adenosine triphosphate (ATP) ATPase activity. When hypoxia persists, there is a metabolic switch to glycolysis, which, for neurons, is a poor metabolic option because of low stores of glucose in brain tissue and the deficient ATP output by the glycolysis pathway. Glycolysis culminates in the generation of lactate, which then accumulates in extracellular compartments, causing acidosis, although it has also been suggested that lactate is an energy source for neurons [23]. Zheng and Wang [24] investigated the regulatory mechanisms of energy metabolism in neurons and astrocytes in the basal ganglia of a neonatal hypoxic–ischemic brain injury piglet model. Their results showed that lactate levels had peaked at 2–6 h after hypoxic–ischemic injury, and glucose peaked at 6–12 h. The expression levels of monocarboxylic acid and glucose transporter proteins (MCTs and GLUTs) increased after HI (peak at 12–24 h) and then decreased. Astrocytes and neuronal damage after HI were not synchronized. These results indicate that lactate and glucose transporters have a synergistic effect on the energy metabolism of neurons and astrocytes following hypoxic–ischemic reperfusion brain injury.

Reoxygenation is another process necessary for survival, but this involves uneven metabolism with certain organs that are metabolically privileged (adrenal medulla, brain, heart), others that are less privileged in this regard (the body in general, kidneys, muscles), and brain regions with irregular metabolism. While reoxygenation occurs, there is an increase in the levels of extracellular glutamate, which enhances activation of Na^+^/K^+^ ATPase to further increase the consumption of ATP. These high levels of extracellular glutamate exceed the buffering ability of astrocytes and produce a continuous overactivation of glutamate receptors. The receptors most severely affected are of the N-methyl-D-aspartate (NMDA) subtype. These reactions intensify Ca^2+^ conductance, leading to improper homeostasis and a condition of metabolic crisis marked by acidosis and an accumulation of lactate, which is reflected differentially during development in distinct areas of the brain. Under these conditions, lactate levels in the neostriatum remain high (>2-fold); however, this does not occur in other zones of the basal ganglia of rats subjected to severe asphyxia when compared to a group of control rats evaluated on postpartum day 8 [25]. This condition can arise in still-immature brains, causing a lesion that can alter the initial plasticity that is required to establish synapses and circuits. In fact, the extra energy the organism consumes in order to reestablish homeostasis could affect the levels it needs to consolidate synapses and circuits. Research based on the Swedish experimental model has identified various alterations that may be associated with perinatal asphyxia (PA). These include thicker pre- and post-synaptic densities, ubiquitination, errors in protein folding, altered levels of synapsin and neurotrophic factors, aggregation, interrupted postnatal neurogenesis, decreased length and branching of neurites, and deficits in myelination. Some studies suggest that plastic changes may occur in an effort to compensate for such neuronal loss. However, compensatory mechanisms, such as over-plasticity, for example, are not necessarily functional and may even aggravate cognitive impairment. Unfortunately, behavioral deficits inevitably and irreparably accompany this insufficiency of neural circuits [26].

Perinatal brain injury can affect infants born at any gestational age; however, preterm fetuses (born < 32 weeks of gestation) are less equipped to adapt to perinatal insults as term infants, making them more predisposed to brain injury [27].

Perinatal asphyxia remains a significant cause of neurological morbidity and mortality in the newborns [14], comprising a decrease in gas exchange, leading to a deficit of O_2_ (hypoxemia) and excess CO_2_ (hypercapnia), with consequent metabolic acidosis. If the episode is prolonged, blood flow and oxygen delivery to tissues are reduced (ischemia) [28]. This condition can have significant repercussions for the neonate, mainly at the level of the central nervous system [29]. 

**Figure 1:**
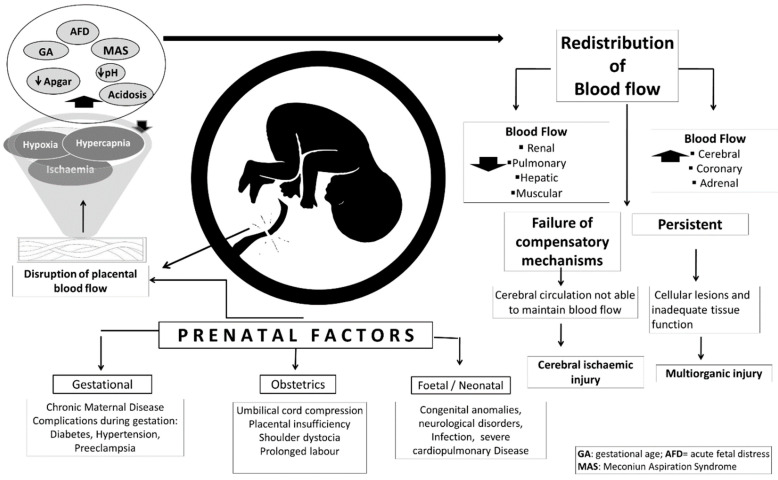
Prenatal risk factors and effect on cerebral ischemic injury.

Fetuses can suffer asphyxia at any point before, during, or after parturition [14]. In terms of frequency, studies indicate that around 50% of cases occur prepartum, 40% during birthing, and the other 10% immediately postpartum [30,31,32,33]. The fact that asphyxia has also been associated with various risk factors during gestation makes the pathophysiology of this condition exceedingly complicated. Risk factors identified to date include chronic diseases in the mother (preeclampsia, hypertension, diabetes) and her age, as these can restrict fetal blood flow and alter placental vasculature [14] (Figure 1), two processes associated with parturition and prolonged labor. Risk factors related to the placenta, meanwhile, include detachment, fetal–maternal hemorrhaging, inflammation, and insufficiency. In addition, the umbilical cord may become occluded, the fetus may develop an anomaly or malformation, intrauterine growth may be retarded, and neurological disorders or spinal cord injuries, among other conditions, may occur [14,34].

Birth in all mammal species is accompanied by a period of obligatory or transient asphyxia in the newborn [30,31,32]. However, the severity of hypoxia–ischemia during these events can be affected by different factors related to the dam, placenta and/or fetus, or the neonate. Asphyxia can occur during fetal life, before, during, or after birth [14]. Approximately 50% of asphyxia occurs before delivery, 40% during parturition, and the remaining 10% during the neonatal period [33].

The pathophysiology of asphyxia is extraordinarily complex and related to several gestational risk factors, such as the dam’s age and chronic maternal diseases, including diabetes, hypertension, or preeclampsia, that can affect the placental vasculature and decrease fetal blood flow [14] (Figure 1) associated with the process of parturition and prolonged labor. Placental risk factors are detachment, fetal–maternal hemorrhage, placental insufficiency, or inflammation. Other fetal neonatal factors involve occlusion of the umbilical cord, fetal anomalies, malformations, and intrauterine growth retardation, as well as spinal cord injuries and neurological disorders, among others [14,34].

### 4.1. Physiological Changes during Birth Asphyxia

Asphyxia markedly alters the physiology of the transition from the intrauterine to extrauterine life. Under conditions of placental hypoxia, circulatory and non-circulatory changes occur in the fetus. From the circulatory changes, the most important mechanism is the centralization of blood flow, or diving reflex, which consists of concentrating blood flow in vital organs, such as the brain, myocardium, and adrenal glands, reducing flow to less essential organs, such as skin, kidney, intestines, and muscle [35]. It is proposed that this mechanism is originated by the chemoreceptor of the carotid body that produces the release of catecholamines that are responsible for centralizing the blood flow; in turn, there is also a decrease in cerebral vascular resistance in order to improve cerebral perfusion. This mechanism of redistribution of flow has limits, and when its severity or duration exceeds these limits, a compromise of the blood flow begins to the encephalon and myocardium, predisposing neuronal damage. Although this mechanism of centralization can ideally preserve fetal physiology in hypoxic conditions, there is evidence that in some neonates, these mechanisms do not develop normally, which can favor brain damage [35].

At the respiratory level, after 30 s of absolute hypoxia, there is a brief period of rhythmic and rapid breaths, which leads to a brief stage of primary apnea (30–60 s). During this phase, there is also bradycardia followed again by panting breaths lasting 4 min after which there is again a secondary apnea, and in the lack of resuscitation, death follows [14,35].

Other mechanisms that attempt to compensate for this oxygen deficiency occur at the level of fetal hemoglobin, which increases its transport capacity and dissociation of oxygen. Additionally, the brain can use other energetic substrates under hypoxia to remain functional, such as lactate and ketone bodies. This is because neuroglial glycogen stores are depleted very quickly (<5 min) [14]. In some cases, neonates successfully recover from hypoxic episodes; however, in prolonged hypoxia, the redistribution of cardiac output causes dysfunction and multiorgan injury [36], leading to hypoxic–ischemic encephalopathy (HIE). Survivors of moderate-to-severe HIE show a high incidence of permanent neurologic and psychiatric disorders, including seizures, cerebral palsy, cognitive delays, motor disabilities, visual loss, hearing loss, behavioral alterations, schizophrenia, and epilepsy [30,37,38].

Basic and clinical research on asphyxia at birth has been supported by animal models, which contribute to the understanding of pathophysiology and lead to the discovery of biomarkers to accurately detect rapid alterations of biochemical pathways [39,40,41,42] in response to the hypoxic environment, as well as the severity of the injury by HIE, allowing for intervention and timely therapy. It is important to understand that the physiology of the fetus differs in fundamental ways from that of the newborn, and those distinctions are both structural and functional in nature. Ensuring the survival of neonates requires several well-orchestrated steps during the passage from intra- to extrauterine life, but these are complex and occur rapidly. Therefore, all caregivers handling newborns need to be well-versed in the physiology of this crucial transition; otherwise, they may fail to perceive deviations from normal physiology or to respond adequately when such scenarios arise. Precisely because asphyxia alters the physiology of transition, managing affected newborns must be based on a thoughtful approach [21].

Human and animal newborns are extremely vulnerable to hypoxia during and immediately after labor as a result of different anatomical, physiological, and biochemical factors. Several mechanisms can be responsible for the lack of fetal or newborn oxygenation: (a) fetal asphyxia due to obstruction of the blood supply in the umbilical cord, for example, torsion or tight circular cord; (b) imbalances of the placental exchange of oxygen, for example, premature placental detachment or retroplacental hematomas; (c) placental perfusion imbalances, for example, maternal hypotension, dehydration, cardiopathies, or severe maternal anemia; (d) failure of the expansion or ventilation or in the pulmonary perfusion of the newborn (asphyxia after birth is usually produced by the latter mechanism, since pulmonary ventilation does not adequately occur) [40,41,42,43,44,45,46,47,48,49]. 

**Figure 2:**
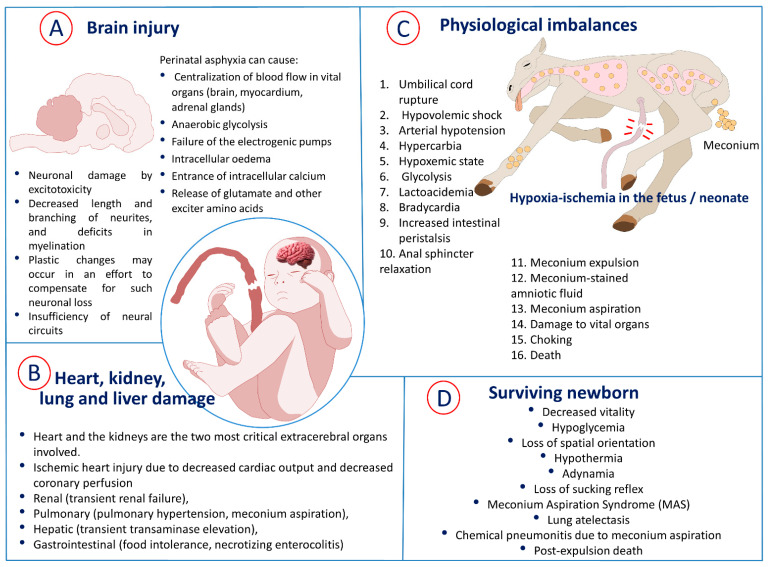
Fetal asphyxia in human and non-human animals, organ injury and physiological imbalances in response to hypoxia–ischemia. Image (**A**) summarizes the neuronal damage at a biochemical and pathological level. Image (**B**) shows damage in other vital organs. In image (**C**), the physiological imbalances are appreciated due to the drastic reduction of oxygen to the fetus, and promote the expulsion of meconium. In letter (**D**) some adverse effects if the newborn survives.

Current clinical criteria of perinatal hypoxia require the presence of four conditions: (1) evidence of metabolic acidosis detected in a sample of umbilical cord arterial blood at birth (pH less than 7.00 and base deficit higher than 12 mmol/L); (2) early onset of moderate or severe encephalopathy in newborns of 34 weeks or more from gestation; (3) cerebral palsy of the spastic or dyskinetic type; and (4) exclusion of other etiologies, such as trauma, sepsis, and coagulopathy, among others [14]. During perinatal asphyxia, several physiologic changes occur in an attempt to compensate for the deficiency of oxygen supply in the newborn (Figure 2).

### 4.2. Mechanisms of Neuronal Injury in Perinatal Asphyxia

Once the compensation mechanisms mentioned above are overcome, a cascade of biochemical events begins in the brain, which eventually leads to different degrees of neuronal and cerebral injury.

As soon as the demands of cerebral O_2_ are not adequately satisfied, the cerebral metabolism begins to perform anaerobic glycolysis with an increase in lactate levels (favoring the process of acidosis) and therefore a reduction in the formation of ATP. This energy deficit affects, in the first instance, the sodium/potassium membrane ATPase pumps, are involved in the maintenance of the membrane potential at neuronal rest [45,46,50,51].

This failure of the electrogenic pumps leads to a secondary accumulation of intracellular sodium, which induces intracellular oedema and loss of the membrane potential at rest, leading to the entrance of intracellular calcium, which favors the release of glutamate and other exciter amino acids, which, in turn, could be involved in the dispersion of neuronal damage caused by excitotoxicity [50]. The entrance of neuronal calcium also activates diverse calcium-dependent enzymatic systems (lipases, proteases, caspases), which are responsible for carrying out proteolysis of cytoplasmic components, also inducing mitochondrial damage. This mitochondrial damage, in turn, produces an increase in oxygen free radicals, which also enhance damage to macromolecules, such as the peroxidation of lipids, proteins, carbohydrates, and nucleic acids [52]. Additionally, all these processes of damage produce the release of inflammatory mediators by the microglial cells, leading to the recruitment of inflammatory cells from the periphery, interstitial oedema and alterations in the permeability of the blood–brain barrier [52].

All of these mechanisms are closely related, and all contribute, to a greater or lesser extent, to primary and secondary neuronal and brain injury. Additionally, it is also widely described that delayed restoration of oxygenation and perfusion to brain tissue can generate even more damage due to previously developed alterations in the permeability of the blood–brain barrier, as well as biochemical changes. This late reperfusion can increase inflammation, the generation of oxygen free radicals, and the interstitial oedema of brain tissue, which can increase final brain damage [14]. 

Despite the fact that at birth, the immune system has not fully matured, it does have the capacity to react to certain kinds of stimuli. When the condition called neonatal hypoxia–ischemia occurs, immune cells in the blood, brain, and peripheral organs are activated and trigger complex, dynamic interactions among these corporal regions. During or after HI, innate and adaptive immune cells in the bloodstream are activated and proliferate, and some extravasation into the parenchyma of the brain occurs. In addition, peripheral immune organs, such as the spleen, may be activated. This seems to exacerbate the insult, since performing splenectomies has been shown to provide some neuroprotection. However, we still do not know exactly how or to what extent specific innate or adaptive immune cells participate in neonatal cerebral lesions. We do know that excessive local cerebral inflammation has detrimental effects, but recent evidence suggests that microglia activation post-lesion may have a protective effect. Another important factor to be considered is the complex interaction that occurs between two systems: the central nervous (CNS) and the peripheral immune system. Responses by the CNS can be modulated by peripheral immune cells and mediators that penetrate the brain; however, at the same time, induction of tolerance and immunity can be orchestrated by brain-derived antigens that drain to peripheral lymph nodes [53].

Finally, because of the activity from all these mechanisms of neuronal damage, diverse strategies of neuroprotection have been implemented with different therapeutic targets (antiexcitotoxic, antioxidant, anti-inflammatory) to limit the neuronal damage associated with perinatal hypoxia and to improve the functional prognosis of the newborns. A complete review of these topics may be found in several recent publications [54,55].

## 5. Cardiovascular Alterations and Multiorgan Dysfunction

As a consequence of asphyxia and ischemia, multiple biochemical mechanisms are responsible for the deterioration of different organs and systems (central nervous system, 28%; cardiovascular system, 25%; kidneys, 50%; and lungs, 23%) [56]. However, the cardiovascular system and hemodynamic instability due to hypoxia, either in the uterus or during the transition of the newborn, have received considerable research attention [36].

During oxygen deprivation in the event of fetal hypoxia–ischemia, compensatory mechanisms are responsible for redistributing cardiac output, centralization of blood flow to vital organs, and reducing oxygen consumption [57]. Although hypoxia–ischemia can affect other tissues and systems, such as the renal (transient renal failure), pulmonary (pulmonary hypertension, meconium aspiration), hepatic (transient transaminase elevation), and gastrointestinal (food intolerance, necrotizing enterocolitis) systems, the heart and the kidneys are the two most critical extracerebral organs involved [56,58]. In a study carried out by Hankins et al. [59], they observed that low oxygen level was not the cause of hypoxic–ischemic encephalopathy (HIE) but that it was a condition developed secondarily to renal, hepatic, and cardiac dysfunction after asphyxia at birth (Figure 2).

### 5.1. Cardiovascular Response

Cardiovascular response consists of transient fetal bradycardia, systemic hypertension, peripheral vasoconstriction, and centralization of blood flow triggered by the release of catecholamines derived from the stimulation of chemoreceptors of the carotid sinus, which detect hypoxemia and transmit afferent impulses to the cardiovascular center in the medulla, then send parasympathetic efferent discharges and peripheral α- and β-adrenergic to the heart, peripheral vasculature, and adrenal glands [14,60,61]. If hypoxia persists, redistribution of blood flow to vital organs is maintained by peripheral vasoconstriction mediated by circulant vasoconstrictors norepinephrine, arginine, vasopressin, neuropeptide Y, and angiotensin II [62]. 

Hypoxia affects myocardial contractility and relaxation, causing hypotension; thus, babies require aminergic support [63]. Hypotension is a common problem found by neonatologists [64]. Asphyxiated newborns are known to have an increased risk of ischemic heart injury due to decreased cardiac output and decreased coronary perfusion [65]. The presence of high levels of cardiac enzymes diagnoses cardiac injury; however, the immediate and long-term structural consequences are not well known, since reported histological findings are minimal [66].

Other pathologies, such as cardiomegaly, have been described [67], including electrocardiogram abnormalities, signs of myocardial ischemia, arrhythmias, atrioventricular valve dysfunction, sustained sinus bradycardia, and decreased ventricular contractility [68,69]. 

Ikeda et al. [70] reported evidence of necrosis with phagocytosis in hearts of lambs with severe asphyxiation [71].

However, although blood supply to the heart is prioritized during an hypoxic event, studies reported deficits in the cardiac function after asphyxia at birth [72]. Sehgal et al. [65], indicate that when redistribution of cardiac output fails to maintain myocardial oxygenation, depletion of cardiac glycogen, anaerobic respiration, and metabolic acidosis occur, and without intervention, continuous circulatory deterioration will occur, eventually leading to myocardial dysfunction, circulatory shock, right and left ventricular insufficiency, tricuspid regurgitation, hypotension, and eventual cardiac arrest. In newborns surviving an intrapartum asphyxia event, the multiorgan damage that may result represents a high risk for the development of severe morbidities throughout life [66].

### 5.2. Renal, Hepatic, Pulmonary, and Gastrointestinal Injury

The reduction in blood supply to the kidney due to the blood redistribution to the peripheral organs to maintain the cerebral, cardiac, and adrenal perfusion during an hypoxic episode allowed for the recognition of Acute Kidney Injury (AKI) as an inevitable consequence of intrapartum asphyxia [66]. It is estimated that between 50 and 72% of asphyxiated newborns with an Apgar score ≤ 6 at 5 min show signs of renal compromise [73]. Cells of renal parenchyma have a limited capacity under anaerobic conditions and a high susceptibility to injury by reperfusion. Saikumar and Venkatachalam [74] suggest that in hypoxia/reoxygenation in vitro models, apoptotic cell death can occur during reoxygenation as a consequence of the mitochondrial release of cytochrome c during hypoxia. 

The decreased excretory function of the kidney, which leads to a reduced capacity to filter blood and maintain blood volume, electrolyte levels, and acid-base homeostasis [75], correlates positively with risk of morbidity and mortality in the asphyxiated newborn [68]. If the kidney injury exacerbates, other organs, particularly the brain, will be damaged [76]. Regarding liver injury, Beath [77], points out that it is probably the cause of hypoperfusion secondary to hypoxia, rather than asphyxia itself. It has been seen that an increase in transaminase levels has some correlation with the severity of perinatal asphyxia [78]. However, Gluckman et al. [79] reported a weak relationship in the transaminase levels in a model of HIE in piglets.

The lungs are organs also commonly affected by asphyxia, so many babies require mechanical ventilation at birth [36]. Pulmonary hypertension, hemorrhage, and significant coagulopathy are frequently observed complications [80]. Concerning gastrointestinal complications, such as necrotizing enterocolitis, are described in infants with asphyxia; however, they are not common. The study of brain injury after intrapartum asphyxia has been the focus of much fundamental scientific and clinical research. When compensatory mechanisms are exceeded and cerebral blood flow can no longer meet demand, a cascade of molecular reactions and mechanisms related to calcium flow, free radical formation, free iron accumulation, and nitric oxide production begins, which leads to cell death [81]. The degree and location of brain injury may vary depending on the type and duration of injury, gestational age, and whether the baby was treated with hypothermia. Studies in both humans and animals have suggested that intermittent asphyxia for less than one hour is not likely to cause brain injury, but severe total asphyxiations can cause brain injury much earlier [82,83]. Brain injury after asphyxia is hypoxic–ischemic in nature, and the neurology injury patterns include selective neuronal necrosis, parasagittal cerebral injury, periventricular leukomalacia, and focal ischemic necrosis [14]. The adverse effects of an hypoxic–ischemic process in the fetus are summarized in Figure 2: both organic damage (A,B) and physiological imbalances (C), as well as some adverse effects if the newborn survives (D).

## 6. Meconium Aspiration Syndrome

Meconium-stained amniotic fluid (MSAF) results from the passage of fetal intestinal content to the amniotic fluid, which occurs with an incidence of 15 to 20% in term pregnancies and 30 to 40% in post-term pregnancies [84]. Among MASF newborns, 3 to 12% may develop meconium aspiration syndrome (MAS) [85]. When a neonate aspirates meconium during intrauterine panting or with the first breaths at birth, MAS develops [86]. MAS is characterized by respiratory distress (from mild tachypnea to severe respiratory failure), which occurs in newborns as a result of bronchoalveolar aspiration of meconium [85]. Among perinatal dysfunctions, MAS is considered a consequence of fetal distress and one of the predominant causes of morbidity and mortality in term infants [86], with important sequelae in lungs and neurologic development at short and long term [44,87]. Its association with asphyxia and pulmonary hypertension is well recognized [85]. It is worth considering that the incidence of MAS, pathophysiology, prevention, and management have changed in the last 20 years.

**Figure 3:**
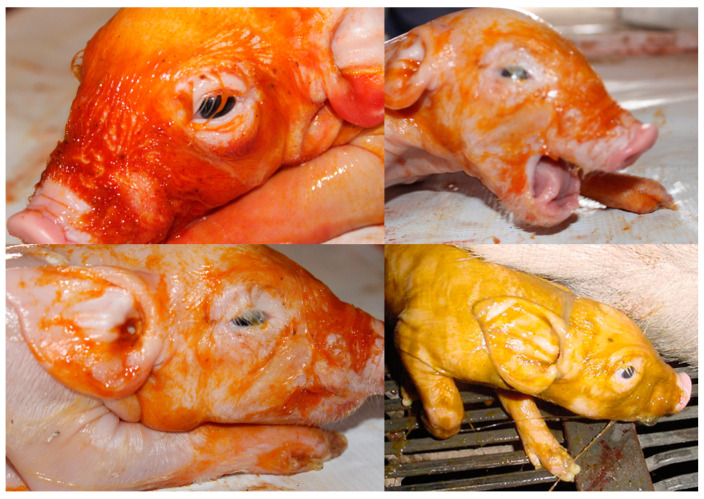
Neonatal piglets with perinatal hypoxia syndrome. When the neonate goes through a severe hypoxia process, it displays bradycardia, yellow to greenish meconium staining, tachypnea, lactic acidemia, hypothermia, hypoglycemia, adynamia, and flaccid muscle tone. Neonates with this perinatal syndrome do not recover, since they do not connect with the teat and are sluggish, which is why they die in the following postpartum hours. There is no neonatal intensive therapy area on pig farms.

Meconium is mainly composed of water (70–80%), as well as intestinal epithelial cells, squamous cells, lanugo, amniotic fluid, bile acids and salts, phospholipase A2, interleukin-8, mucous glycoproteins, lipids, and proteases [48] (Figure 3). Meconium is present for the first time in the fetal intestinal tract between 70 to 85 days of gestation. It is recognized that the passage from meconium to the amniotic fluid is due to fetal gastrointestinal maturity or fetal stress secondary to hypoxia and infection. However, the pathophysiology of MAS is very complex and not yet fully understood. Fundamental questions arise, as some neonates exposed to MASF develop MAS, while others do not [88].

It is known that several mechanisms are implicated in the pathophysiology of MAS, including acute airway obstruction (considered the primary mechanism of MAS in the past), surfactant dysfunction, chemical pneumonitis and the direct toxic effect, and persistent pulmonary hypertension of the newborn (PPHN) with a right-to-left shunt and secondary infection. Other authors suggest that fetal systemic inflammation is also a risk factor for the development of MAS in newborns with MSAF [89,90,91].

Despite the complexity of the passage of meconium to the amniotic fluid, what is certain is the damage it generates when it is aspirated, such as airway obstruction, air trapping, hyperinflation of the lungs and subsequent pneumomediastinum or pneumothorax, partial alveolar collapse, and complete obstruction of the smaller airways, which may cause atelectasis [44,51,91,92,93]. Additionally, pneumonitis can develop due to the direct toxic effect of meconium components and the infiltration of a large number of polymorphonuclear leukocytes and macrophages. A decrease in lung capacity and oxygenation has been reported as a result of a reduction in surface tension of the surfactant due to meconium [84].

## 7. Criteria for Diagnosis of Hypoxia–Ischemia

Animal studies in newborns are crucial for our understanding of transitional physiology and current resuscitation practice; as Dawes showed in the sixties [93], sustained hypoxia in utero, during labor or postnatally, manifests as an increased or absent respiratory effort, followed by a period of apnea (primary apnea). During this period, heart rate falls, but blood pressure is maintained. If asphyxia continues the infant gasp and the heart rate and blood pressure fall, and secondary apnea appears with anaerobic metabolism, lactic acidosis and myocardial performance compromise [94]. Ventilation is required to recover positive pressure, and if the insult lasted long enough, cardiac compressions are needed. Hypoxic–ischemic encephalopathy is the clinical manifestation of generalized disordered neurologic function due to hypoxia. In HIE, as opposed to other etiologies of neonatal encephalopathy, criteria for its diagnosis include (in neonates ≥ 35 weeks gestational age) [95]: evidence of intrapartum hypoxia (significant hypoxic or ischemic event immediately before or during labor or history consistent with fetal heart-rate compromise) and two or more of the following:

a. Apgar score of <5 at 5 and 10 min;
b. Need for mechanical ventilation or resuscitation at 10 min;
c. Acidemia documented in fetal umbilical artery (pH < 7.0 or base deficit ≥ 12 mmol/L);
d. Multisystem organ failure;
e. Evidence of moderate or severe encephalopathy staging, often supported by neuroimaging with evidence of acute brain injury consistent with hypoxia–ischemia.

### Clinical Assessment

Clinical assessment and disease staging are based on the scale developed by Harvey and Margaret Sarnat [96]. Frequently, a modified (simplified) score, such as Thompson’s, is used [97]. Moderate and severe grades of encephalopathy have a poor prognosis, as opposed to mild. Therefore, two combinations of signs have been used as criteria [79,98], including:

1. Reduced responsiveness with hypotonia or incomplete reflexes (including weak suck) or clinical seizures.
2. At least three signs from the following categories:
  (a) Reduced responsiveness;
  (b) Reduced activity;
  (c) Abnormal posture;
  (d) Abnormal tone;
  (e) Incomplete reflexes;
  (f) Abnormal pupil response, heart rate, or respiration.

## 8. Neonatal Hypoxic–Ischemic Encephalopathy: Clinical Aspects

Neonatal hypoxic–ischemic encephalopathy (NHIE) in babies born at term or preterm may occur at different periods of the newborn’s life. These periods are antepartum, intrapartum, and postpartum. NHIE puts the neonate at a higher risk of suffering visible neurological alterations [99].

Neonatal hypoxic–ischemic encephalopathy, as a clinical syndrome, was described in the second half of the twentieth century and included: (1) evidence of fetal stress based on records of heart rhythm and amniotic fluid stained with meconium; (2) signs of depression in the neonate at birth; (3) neurological syndrome progressing in the first hours/days of life [100]. The clinical features of NHIE from childbirth and after 12 h include low levels of consciousness, for example, stupor or coma; a respiratory abnormality, such as periodic breathing or failure; intact pupil response; hypo or hypertonia; and seizures.

Clinical characteristics from 12 to 24 h of life are stupor–coma or some other alert impairment, more seizures, apnea events, and weakness (in proximal limbs or hemiparesis in full-term infants; in lower limbs in premature infants) [101]. 

From 24 to 72 h, infants show stupor–coma, respiratory arrest, abnormalities of the oculomotor brain stem and pupil, deterioration of consciousness after a hemorrhagic infringement in full-term infants, or interventricular hemorrhage in preterm infants [101]. 

After 72 h, the stupor–coma remains persistent, as well as abnormal movements of suckling and swallowing, hypo and hypertonia, and weakness [101].

Some pathological brain changes involve [102] selective neuronal necrosis of the cortex, basal ganglia, thalamus, brain stem (reticular formation and other nuclei), cerebellum, and anterior horns from the spinal cord.

**Figure 4:**
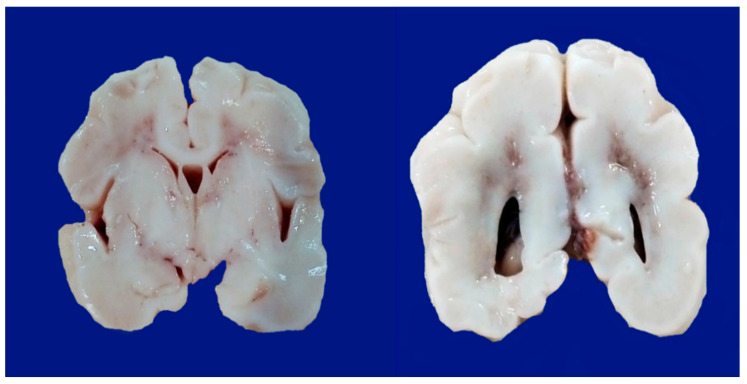
Periventricular white matter greyish discoloration and edema in a male, 32 weeks of gestational age old, with HIE. (Courtesy: María de Lourdes Cabrera-Muñoz, MD, Department of Pathology, Hospital Infantil de México Federico Gómez).

**Figure 5:**
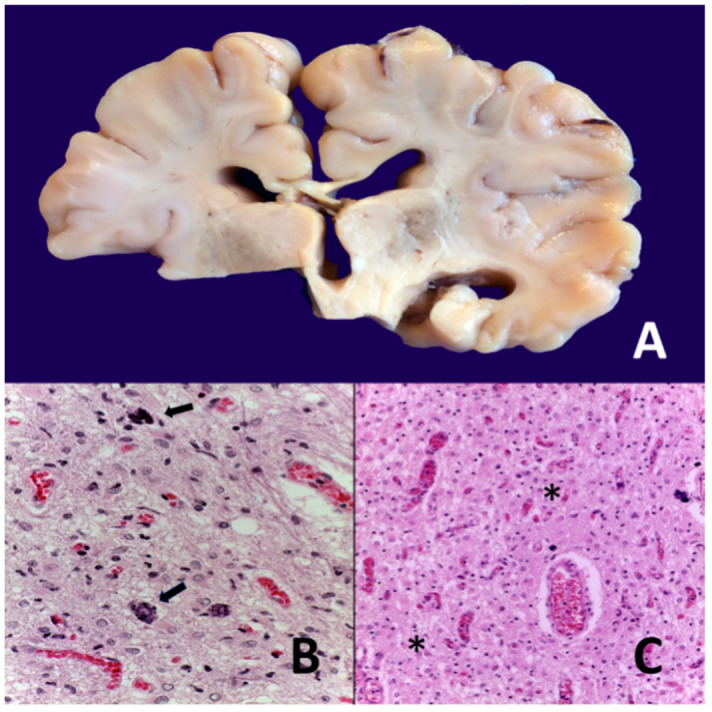
(**A**). Bilateral basal ganglia necrosis in a male at 39.6 weeks gestation with severe perinatal asphyxia secondary to congenital heart disease. (**B**). Neuronal necrosis and calcification (arrows). (**C**) Withe matter infarct (*) HE 40X. (Courtesy: María de Lourdes Cabrera-Muñoz MD. Department of Pathology, Hospital Infantil de México Federico Gómez).

- Parasagittal injury of the cerebral cortex, in subcortical white matter in the lateral convection of the superior-medial orientation, in the posterior–anterior direction.
- Periventricular leukomalacia with necrosis in the subcortical white matter of the hemisphere, including descending motor fibers, optical radiations, and association fibers.
- Focal and multifocal necrotic ischemia in the cerebral cortex and subcortical necrosis in white matter, mainly unilateral with a vascular distribution.
- Unilateral brain lesion with the presence of minor injury in the contralateral hemisphere. The most frequent lesion is in the area of irrigation of the medial cerebral artery: cerebral cortex, white matter, basal ganglia, and posterior limb of the internal capsule (Figure 4 and Figure 5).

However, the prevention of brain damage or the use of neuroprotective drugs in animal models was not successful as expected. More research is necessary for the benefit of newborns suffering NHIE in order to avoid neurological sequelae and development deviations.

### 8.1. Inflammatory Biomarkers of Birth Asphyxia

Understanding the causes of asphyxia and the intermediate steps between hypoxia and fetal death can allow for the identification of biomarkers that enable prediction and prevention of fetal death, mainly in women at risk of this clinical complication [103]. 

Several biomarkers have been studied. Creatinine, liver enzymes, cardiac troponin I, prolonged coagulation times, and thrombocytopenia help to address multiorgan dysfunction. In one study, lactic dehydrogenase (LDH) sampled within 6 h of birth was found to provide prognostic information: <2085 U/L survived without disability vs. 3555 U/L (interquartile range 3003 to 8705) who were disabled [104]. 

In the last decade, the search for useful biomarkers to accurately predict the severity of the lesion in perinatal asphyxia and HIE has become an area of growing interest in neonatal research [38]. However, despite the potential of many promising markers, few studies have been validated or used in clinical practice [105]. 

### 8.2. Placental Inflammatory Biomarkers

It is well known that the placenta mediates the interactions between the mother and the fetus during pregnancy. Thus, in a clinical study with a population of neonates with high-risk evidence, with acidosis and encephalopathy, placentas were analyzed, and 95% were found to have abnormalities. Additionally, 65% met the criteria for diagnosis of an important placental pathology, with a high incidence of inflammatory processes (chorioamnionitis and chronic patchy/diffuse villitis), which were significantly associated with abnormal outcomes of neurological development two years after treatment with hypothermia. Therefore, these findings suggest an important contribution of placental inflammatory mechanisms in the severity of asphyxia [106,107].

### 8.3. Serum Brain Biomarkers

Within the neuronal biomarkers detected in the serum of neonates with HIE we can find glial fibrillary acid protein (GFAP), which is an intermediate filament protein released from astrocytes [108]; ubiquitin carboxyl-terminal hydrolase (UCH-L1), a specific cytoplasmic neuron enzyme and a marker for neuronal apoptosis that is concentrated in dendrites [108]; S-100B, a calcium-binding protein released in astrocytes; neuron-specific enolase (NSE), a glycolytic enzyme in neurons [109]; ubiquitin carboxy-terminal hydrolase L1 (UCH-L1); or total tau protein, which indicates astrocytic and neuronal damage. These proteins are released into the blood through the increased permeability of the blood–brain barrier [81].

Another characteristic of traumatic brain injury is an imbalance between oxygen delivery to the brain and consumption. Post-insult alterations in the flow rate and volume of oxygen in the blood and arteries can immediately compromise the delivery of oxygen to the brain. When this occurs, the area around the lesion suffers an hypoxic condition that affects brain resident cells and infiltrated neutrophils. Various published reports mention that under hypoxic conditions, microglia can release nitric oxide and a series of cytokines. This sustains neutrophil activation, triggering persistent hypoxia and neuronal death [110]. Sorokina et al. [111] suggested the involvement of nitric oxide and its products in immune responses and that traumatic brain injuries (TBI) may be accompanied by nitrosative stress. Regarding the first two days of mild-to-moderate vs. severe TBI, these authors suggested that the opposed natural levels of NR2 (NMDA) antibodies could be related to a key mechanism that operates to protect neurons from Glu excitotoxicity.

In theory, biomarkers can identify at-risk pregnancies and allow a proper intervention before an irreversible lesion occurs in the fetus due to hypoxia; however, many studies, although novel, are small pilot tests that have no power to predict long-term results. Therefore, it is necessary to continue researching this topic [81,112]. Several biomarkers have recently been isolated that may aid in identifying neonatal neurologic injuries in the immediate postpartum period. Trials with 10-day-old mice showed that the HI condition significantly increases osteopontin significantly, but that this did not occur after administering LPS. The mRNA of osteopontin was induced in the brain but not the blood. Since immunostaining showed the expression of osteopontin by the microglia/macrophages in brains lesioned by HI, the authors suggested osteopontin as a possible prognostic blood biomarker in cases of HIE related to the activation of microglia in the brain. They further posited that an increase in osteopontin in the bloodstream may be indicative of a perinatal event that occurred up to 24 h earlier. Hence, osteopontin might be an effective marker of previous HI stress in the uterus and of an inadequate response to treatment for hypothermia [112].

The most reliable approach for identifying and evaluating the seriousness, timing, and pattern of these kinds of insults may well consist in measuring an array of inflammatory and neuronal biomarkers at a precise point of care at various intervals. Full pathway analysis employing various omic strategies could lead to the identification of new therapeutic targets for the treatment of neonatal HI cerebral lesions. However, research into neonatal brain biomarkers is still at its outset, so we may have to wait some time for major advances in this area [113].

### 8.4. Electrophysiology

Amplitude-integrated electroencephalography (aEEG) has been widely used in neonatal intensive care units (NICU) and can help to diagnose encephalopathy by the bedside. It provides an objective record that can be reviewed by a pediatric neurologist if needed. In mild forms of encephalopathy, the background pattern might change from continuous to discontinuous. This is called discontinuous normal voltage and does not represent an important abnormality. If the injury is more severe, a low-voltage background with periods of normal amplitude might be seen; this is called burst suppression. On more severe disturbance is when there is no burst, only continuous low-voltage activity that can progress to a flat trace. Burst suppression, continuous low voltage, and flat trace are severely abnormal. Spitzmiller et al. [114] found in metanalysis (8 studies) that aEEG has an overall sensitivity of 91% (95% CI 87–95%) and a negative likelihood ratio of 0.09 (95% CI 0.06–0.15) in prediction of poor outcomes [114].

### 8.5. Near-Infrared Spectroscopy

**Figure 6:**
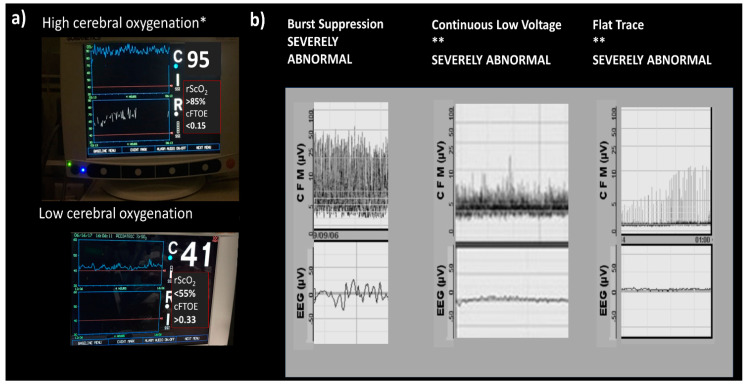
(**a**) High cerebral oxygenation on NIRS * with (**b**) a low-electrical-activity aEEG background ** in severe HIE neonates on hypothermia treatment at 12 h of age has a 91%positive predictive value for long-term adverse neurological outcome (magnetic resonance imaging and neurodevelopmental assessment at 18 months of age), and the absence of these results in a negative predictive value of 100%. NIRS: near infrared spectroscopy; aEEG: amplitude-integrated electroencephalography; rScO_2_: regional cerebral oxygenation; cFTOE: cerebral fractional tissue oxygen extraction. Courtesy: Daniel Ibarra-Ríos, MD, Department of Neonatology, Hospital Infantil de México Federico Gómez.

Regional cerebral saturation (rScO_2_) and fractional tissue oxygen extraction (cFTOE) have also been used to monitor oxygen delivery to the brain by near-infrared spectroscopy (NIRS). High cerebral oxygenation on NIRS with a low electrical activity aEEG background in severely HIE neonates on hypothermia treatment at 12 h of age has a 91%, positive predictive value for long-term adverse neurological outcomes (magnetic resonance imaging and neurodevelopmental assessment at 18 months of age), and the absence of these results in a negative predictive value of 100% [115] (See Figure 6).

### 8.6. Neuroimaging

**Figure 7:**
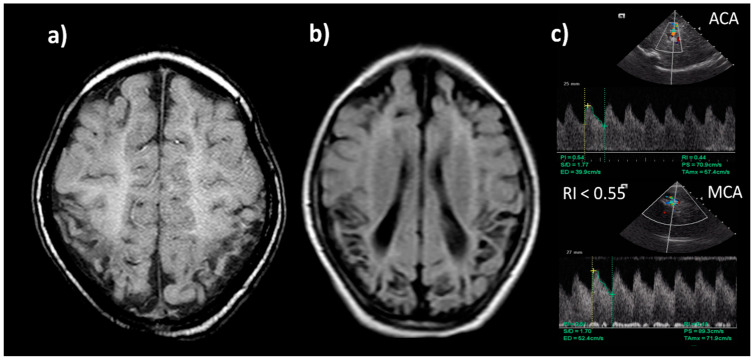
Magnetic resonance: (**a**) axial image of sequence enhanced in T1 towards convexity and (**b**) enhanced in FLAIR, where hyperintensity of the bilateral semioval centers is observed, as well as a decrease in volume in bordering territory in parietal regions and in the smaller frontal portion with retraction of the lateral ventricles associated with hyperintensity of the periventricular white matter. Courtesy: Eduardo M. Flores Armas, MD, Department of Medical Imaging. Cranial ultrasound: (**c**) low RI (<0.55) in normothermic infants or after rewarming has an 84% positive predictive value for death or disability. Courtesy: Daniel Ibarra-Ríos, MD, Department of Neonatology, Hospital Infantil de México Federico Gómez. ACA: anterior cerebral artery; MCA: medial cerebral artery; RI: resistive index.

Cranial ultrasound is useful to document antenatal injury. After 24 h, cerebral vasodilatation can be documented in a low cerebral resistance index (cRI). Low RI is not predictive of poor outcome during hypothermia, but in normothermic infants or after rewarming, a value below 0.55 has an 84% positive predictive value for death or disability [116] (See Figure 7).

Magnetic resonance is the imaging choice for prognosis. Diffusion-weighted imaging helps to detect abnormalities within the first week. A study of between 7 and 21 days is essential to document injury to basal ganglia, the internal capsule, white matter, brainstem and cortex. In conjunction with cranial ultrasound, it can help to detect complications or comorbidities such as infarction, hemorrhage, and malformations. The evolution of diffusion abnormalities on MRI following HIE in term infants on therapeutic hypothermia (current treatment) has been studied [117]. The above led to the development an MRI injury scoring system. Higher MRI injury grades were significantly associated with worse outcomes in the cognitive, motor, and language domains of the Bayley-III [118,119].

### 8.7. Hemodynamic Management

Echocardiography is an important diagnostic tool to delineate two different clinical scenarios [120]:

1. Low systemic blood flow with normal oxygenation: with echocardiographic findings consistent of left ventricle (LV)/right ventricle (RV) dysfunction in which management must include positive inotropes.
2. Low systemic blood flow with impaired oxygenation. In this scenario, the echocardiographic finding can show:
  (a) Persistent pulmonary hypertension, where management should include pulmonary vasodilation, subsequentially augmenting systemic blood flow after pulmonary venous return improves;
  (b) LV dysfunction with PPHN, where management must include positive inotropy and maintenance of right-to-left ductal shunt to support systolic blood flow;
  (c) RV dysfunction with PPHN, where management must include positive inotropy, reduced RV afterload (pulmonary vasodilation and consideration of prostaglandin E1 if the ductus arteriosus is restrictive) and maintenance of adequate RV preload.

Nitric oxide remains the vasodilator of choice. Low-dose vasopressin is a physiologically suited drug in HIE newborns with systemic hypotension and oxygenation failure, as it can produce systemic constriction and pulmonary vasodilation, as well as theoretical stimulation the endogenous production of nitric oxide, as noted in animal models [121]. Milrinone, a widely used inotropic/dilator in neonatology, is not recommended, as drug clearance is lower (especially in patients on therapeutic hypothermia), and might cause extreme hypotension [122].

Hochwald et al. [123] found that neonates with brain injury on magnetic resonance imaging had higher superior vena cava (SVC) flow pre-rewarming compared with newborns without brain injury, possibly reflecting a lack of cerebral vascular adaptation in newborns with more severe brain injury [123]. Is important to note that LVO is lower in patients receiving therapeutic hypothermia (mean ± SD 126 ± 38 mL/kg/min), so one must be cautious interpreting it. Sakhuja et al. [124] found that celiac artery and superior mesenteric artery blood flow velocity and LVO did not vary during hypothermia but rose after rewarming, suggesting a protective effect of therapeutic hypothermia on the gastrointestinal system [124].

Hemodynamic assessment might have prognostic value, as Giesinger et al. [125] found studying 53 patients with HIE undergoing hypothermia. RV dysfunction was associated with risk of adverse outcome; high brain-regional oxygen saturation and low middle-cerebral artery resistive index were associated with RV dysfunction in post hoc analysis [125].

## 9. Targets for Neuroprotection

### Potential Interventions for Birth Asphyxia: A Window for Reducing Further Brain Injury

Studies of animals and humans show that both the stage of development and the seriousness of HI lesions impact the brain’s selective regional susceptibility to damage and, as a result, the clinical manifestations that ensue from such damage. This approach, however, has one serious drawback: the fact that of every six or seven neonates with HI treated with therapeutic hypothermia (TH), only one is saved from death or serious disability by the age of 18–22 months. The HELIX trial, which looked into hypothermia for encephalopathy in economically developing countries with low and middle incomes, was published in 2021, adding complexity. It was a multicenter, open trial performed with a randomized control method. The study, which included 408 newborns, was conducted with a thoroughly strict procedure. For the group with hypothermia, 202 subjects were included, and for the control group, 206. The study concluded that hypothermia in these developing countries did not reduce mortality or moderate-to-severe impairment at 18 months, but it remarkably increased the number of deaths [126]. The lack of hypothermic neuroprotection in developing countries could potentially be associated with the subacute nature of brain injury in these countries, as demonstrated by the pathway of partial prolonged hypoxia (intermittent hypoxic injury) presented in economically developing countries vs. acute hypoxia manifested in high-income countries, as proven by MRI spectroscopy and gene-expression studies [127]. These results indicate that alternative techniques must be developed to increase the efficiency of TH or replace it [128].

Based on different animal models on newborn swine, rat pups, and fetal sheep [129,130], clinical trials of head cooling combined with body cooling and whole-body cooling alone were conducted [131]. As previously mentioned, current evidence from 11 randomized control trials on asphyxiated newborns with 36 weeks of gestation or more found that moderate hypothermia (33.5 °C for the whole body and 34.5 °C for head combined with body cooling) initiated less than 6 h after birth for 72 h with a rate of rewarming of 0.5 °C/h reduces death and neurodevelopmental disability among moderate and severe cases of HIE (64). A recent trial demonstrated that among infants of at least 36 weeks of gestation with HIE, more extended cooling was not superior to 72 h of cooling, and more profound cooling was not superior to cooling to 33.5 °C [132]. Gunn et al. [130] studied a fetal sheep model of hypothermia, cooling at 1.5, 5.5, and 8 h. They found the highest levels of neuroprotection (neuronal loss score) at 1.5 h, less (but favorable) neuroprotection at 5.5 h, and no neuroprotection at 8 h [133]. These findings constitute the basis for the conclusion that hypothermic neuroprotection is time-sensitive. A question that has not been answered is whether, on humans, a wider window of opportunity exists beyond 6 h, which is very important in limited-resource settings where transport to third-level care might take longer. At present, a clinical trial is addressing that question [8]. It is crucial to prevent hyperthermia, as one of the clinical trials showed that the odds of death or disability were quadrupled for each 1 degree C increase in the highest quartile of the skin or esophageal temperatures [134]. Some groups use the term therapeutic normothermia to designate all the measures taken at the bedside to avoid iatrogenic hyperthermia. Another critical question to be addressed is whether there is a benefit of cooling late preterm infants. So far, case reports have shown mixed results, but a lack of safety is an issue. Currently, a clinical trial is recruiting preterm infants of 33 to 35 weeks gestational age who present at < 6 h postnatal age with moderate to severe neonatal encephalopathy [134]. Supportive management for neonatal encephalopathy includes adequate resuscitation, avoiding hyperthermia, maintaining PaCO_2_ in the normal range, adequate hemodynamic management according to clinical scenario, avoiding hypertension, and cautious volume management (initial volume restriction) following serum sodium and fluid balance and serial glucose, with prompt correction when abnormal [120,135]. 

In addition to hypothermia, the most promising adjuvant therapies include Xenon and erythropoietin [135]. Xenon is an anesthetic gas that acts as a non-competitive NMDA antagonist. The largest trial in humans is the TOBY-Xe study, wherein one arm was treated with hypothermia and xenon (administered within 12 h of life for a duration of 24 h vs. hypothermia alone). No differences were found in biomarkers of cerebral damage, and this is believed to be influenced by the late initiation (median 10 h) of the drug [136]. Erythropoietin (EPO) is assumed to work as an antioxidant, an anti-inflammatory, and an inducer of antiapoptotic factors [137]. Protection has been found in stroke and hypoxic–ischemic animal models [138,139,140]. Razak et al. [141], in metanalysis (6 studies 5 with EPO and one with darbepoetin, N; 454), found a reduced risk of brain injury identified in EPO-treated infants, as well as a trend toward a lower risk of death [10].

To date, EPO is the only approach that has been tested in primates, accompanied by follow-up that allows for the calculation of anticipated effect sizes for the combined outcomes of cerebral palsy and death.

With the possible exception of melatonin, none of the therapeutical approaches examined herein necessarily requires intensive care measures. However, additional pre-clinical research on the processes of infection and inflammation are needed before trials can be contemplated [142].

Adverse neurodevelopmental results due to HIE can be reduced by treating (subclinical) seizures and routine EEG neuromonitoring. The large number of patients who suffer undesirable outcomes, however, indicates the need to focus research on complementary therapies and interventions that can improve results. There is an urgent need for funding to carry out studies of this kind. The evidence available at this time supports beginning TH as soon as indicated to obtain optimal results in terms of neuroprotection [143]. Topiramate is a sulfamate-substituted monosaccharide and carbonic anhydrase inhibitor. A substance similar to acetazolamide, it was first analyzed as an anticonvulsant with potential for neuronal repair. It also seemed promising for repairing post-ischemic myocardial damage. The best protection seems to come from treatment with melatonin, a pineal hormone with circadian secretion and maximum nocturnal values. Melatonin has several important abilities, as it can diminish oxidative stress, scavenge free radicals, and improve cellular physiology. Recent stem cell research has raised expectations regarding regenerative medicine, where the functionality of cells damaged by hypoxic insult could be improved or replaced by progenitor cells. The paracrine role of progenitor cells and stimulation of myocardial angiogenesis/repair are reflected in current data [144].

## 10. Neurodevelopment of Babies with Asphyxia

**Figure 8:**
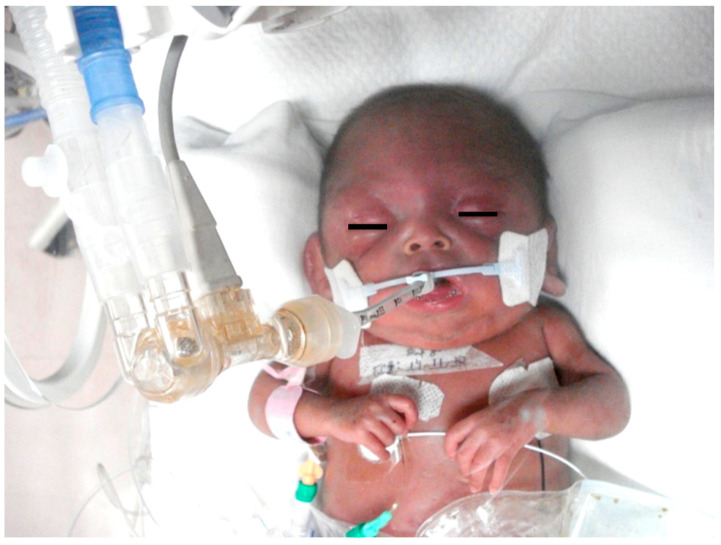
Male of 34 weeks of gestational with multiorgan damage (heart, lungs, liver, gut, and kidneys) after severe perinatal asphyxia. (Courtesy: Dina Villanueva-García, MD, Department of Neonatology, Hospital Infantil de México Federico Gómez).

Deviations in the neurodevelopment of infants after an NHIE event have recently been the focus on many investigations. Researchers have found a large percentage of child survivors of NHIE with significant neurodevelopment sequelae [145] (Figure 8). There is a close relationship between the degree of NHIE and the outcome. When the risk of death was 12.5%, neurological disabilities had a frequency of 14.3%—the more severe the NHIE event, the greater the number and severity of sequelae. Therefore, clinical classification of NHIE has prognostic value [146]. More severe symptoms of neurological dysfunction in the first days of life are associated with a more severe outcome [145].

The main neurological alterations after an NHIE event are cerebral palsy, blindness, deafness, epilepsy, mental retardation, delayed motor control, speech delay, attention deficit hyperactivity disorder, reading–writing disability, and psychiatric alterations. At-risk Infants must be followed by a long interval of time, at least during the first years of the school-age period. If the necessary resources are available, it is recommended to continue monitoring until adolescence and youth [146]. The observation of the findings related to the language of children with NHIE indicates that deficits may become evident only with advancing age and stages of childhood neurodevelopment [147]. The aim is to prevent the development of neurological disabilities, control seizures and abnormal movements, aid in orthopedic development, and promote the development of cognitive functions and brain plasticity. The rational use of therapeutic measures should produce normal or almost normal neurologic development of these at-risk infants. 

Prognoses of the clinical course, severity, and results of any disease are important. In this regard, studies have analyzed organ-specific proteins as potential plasma-based biomarkers of insults. To date, they have been found to be of poor specificity and sensitivity for routine clinical use, but research to identify biomarkers that can characterize brain injury in critically ill children is ongoing [148].

## 11. Animal Models of Perinatal Asphyxia

**Figure 9:**
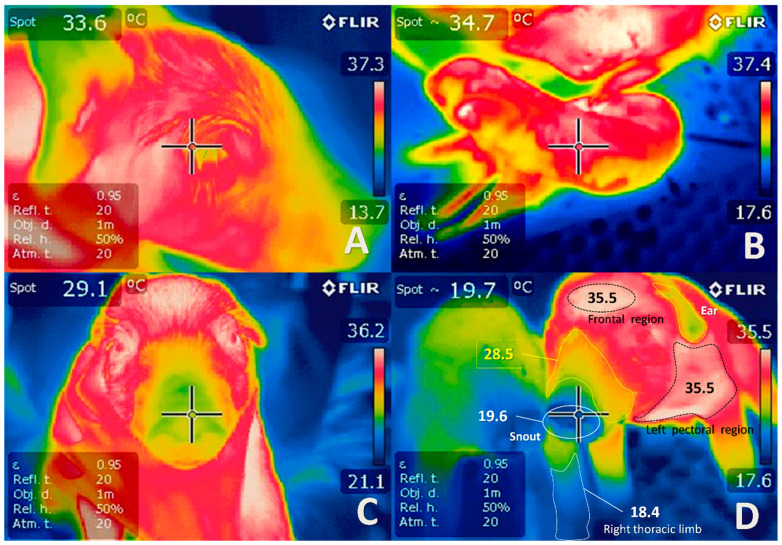
Thermograms of newborn piglets with severe hypoxia and meconium staining on the skin to a severe degree. It is important to dry the newborn immediately, since the humidity of the amniotic fluid favors a rapid drop in body temperature. The areas marked in yellow on the thermograms indicate temperatures between 28 and 32 °C, especially in images (**A**–**C**), where a marked drop in temperature is seen in peripheral areas of the auricular pavilion, thoracic limbs, and especially the face, particularly the snout. In thermographic image (**D**), we can see a piglet stained with severe meconium grade, with umbilical-cord rupture, a failing vitality score, and severe hypothermia, despite having been dried. It is important to note that different temperatures are seen depending on the body region. In the frontal area of the head and left pectoral region, the highest surface temperatures are observed (35.5 °C). In yellow (image (**D**)), the proximal region of the snout and the auricular pavilion (28–29 °C) are distinguished, and in the distal region of the thoracic limbs and the distal snout area (in blue), the lowest temperature ranges of 18–19 °C are shown. The use of infrared thermography is essential to understanding the changes in the vascular microcirculation in the study of hypothermia in newborns with asphyxia.

The use of animal models has significantly contributed to the understanding of perinatal asphyxia. Several studies have used animal models for the induction of fetal asphyxia, including maternal hypoxemia, infrared thermography (IRT), reduction of in uteroplacental blood flow, umbilical cord occlusion, and embolization, among others [46,47,48,51,92,147,148,149,150,151,152,153] (Figure 3 and Figure 9). Infrared thermography (IRT) is a technique used in both veterinary and human medicine to quantify the surface temperature of the skin based on visualizations of thermographic changes [154]. The use of IRT is essential to understanding the changes in the vascular microcirculation in the study of hypothermia newborns with asphyxia (Figure 9). In veterinary medicine, thermography has brought several benefits for animal models in terms of evaluating lesions, diseases, and surgical procedures [150,155,156,157,158].

During the last six decades of the study of perinatal asphyxia, various animal species have been included from non-human primates, sheep, pigs, canine, and rodents, among others. 

While it is true that models of small animals with mouse, rats, and rabbits to analyze lesions for neonatal asphyxia/hypoxia have provided relevant data, models of larger animals that use pigs, sheep, and non-human primates have provided additional essential information necessary to initiate clinical trials [159]. For example, piglets have been frequently used for studies of oxidative-stress conditions as a result of the hyperoxic challenge due to the transition from the hypoxic intrauterine environment to extrauterine life [160] (Figure 3). The ovine fetus has been widely used for physiological and pathophysiological studies of the brain [161]. The non-human primate model is unique because of the physiological and anatomical similarities to humans and because neurological development tests adapted from those of humans can be used [159].

However, a benefit of neonatal models with rodents is the advantage that pups exhibit postnatal cerebral development analogous to human development in the third trimester, and litters are easily produced and easy to handle. The main disadvantages of neonatal rodent models are related to their lissencephalic brain organization, a lower proportion of white and grey matter compared to primates, and the limited behavioral repertory. Compared to rodents, the development and complexity of the brain in non-human primates are similar to those in humans, allowing for applications of sophisticated neurological tests over time [162].

## 12. Scientific Findings of Perinatal Asphyxia in Animal Models: Advantages and Limitations

In general, animal models have significantly contributed to the field of neonatal medicine. Specifically, research on animal models has provided the evidence base for the therapeutic treatment of newborns with NHIE [163].

Some animal models that have been proven crucial for perinatal brain research are non-human primate fetuses and neonates, as well as pregnant sheep, lambs, puppies, piglets, and immature rodents. Although no model is perfectly ideal in terms of capturing the diversity and complexity of human brain pathology, the investigator must evaluate the strengths and limitations of each model in the context of the research questions [164].

Clinically, it is challenging to estimate the exact time or duration of hypoxia damage, and even more, neurologic lesions as hypoxic–ischemic injury. However, animal models give us the ability to control the time of the injury, allowing for programmed detection of the alteration of metabolites and clarification of the pathophysiological mechanisms [38].

In the case of sheep, they have been used for a variety of purposes, helping to elucidate, for example, how subjection to moderate hyperglycemia before ischemia for a pro-longed time and during reperfusion does not influence the length of brain injury. On the contrary, immense damage to the fetal brain can result from exposure to an additional acute increase in plasma glucose concentration before ischemia [161].

Other relevant contributions in lambs include the fact that renal tubular injury occurs with all degrees of asphyxia, despite varying degrees of brain injury. Lamb models have also helped to establish a correlation between morphological changes in the myocardium and liver, which were previously only associated with severe brain damage. Therefore, oxidative stress appears to play a role in liver damage pathogenesis (Table 1) [70].

Additionally, there have been successful studies to determine variations in the regional blood flow of fetal sheep under severe asphyxia and presenting neurological impairment (presence of seizures) (Table 1) [165].

On the other hand, preterm piglets present inadequate ventilation, along with clinical risks comparable to the development of respiratory distress syndrome, thus representing a viable alternative to animal models of sheep and non-human primates [168].

Other studies in piglets have aimed to investigate in detail the prospective implications of the success of cardiopulmonary resuscitation and short-time survival, as well as the presence of non-perfusing cardiac rhythms in asphyxiated newborns [169]. Interesting research has helped in the development and validation of a model of birth asphyxia by performing umbilical cord clamping in term piglets during caesarean sections under general anesthesia as an imitation of the progress of birth asphyxia during natural parturition [170]. Other relevant studies and their contributions in piglets are shown in Table 2.

Term rodent animal models are used in basic research on the mechanisms of specific diseases [175]. Baboons and premature lambs provide an idea for clinical applications, and rabbits seem to be the most useful animal models due to their possible application in both situations [176,177], in addition to the fact that their medium size greatly facilitates handling in many investigations, reducing costs [168].

Other studies in rodents have contributed to the study and comprehension of the conditions caused by the model of subchronic perinatal asphyxia, which has proven to be effective for the study of conditions of asphyxia during pregnancy. It is non-invasive, reliable, and easy to replicate, and it also allows for control of the conditions of asphyxia. It appears to be adequate for screening and research on asphyxia indicators in the dam and fetus [178]. Other relevant studies and their contributions in rodents are shown in Table 3.

On the other hand, baboons have been used as animal models since 1980 in research on infectious and cardiovascular diseases, obesity, and hypertension, among others, since the stages of intrauterine development of the lungs, brain, kidneys, and adrenals are very similar to those in human fetuses. However, non-human primates have high economic handling costs, in addition to the fact that, for ethical reasons, their use as animal models for the study of bronchopulmonary dysplasia has been discontinued [183,184,185].

Research in primates has been relevant in the study of perinatal asphyxia, such as the first study in a modified model that aimed to detect thalamic lesions with magnetic resonance, which allowed for the opportune detection of biomarkers related to specific patterns of newborn brain injury that could be useful for the validation of possible treatments of neonatal hypoxic–ischemic encephalopathy [162]. Measurements of the resistive indices at the thalamus level have also been important; they could potentially supplement other measures for anticipating results from the population of infants with hypoxic–ischemic encephalopathy [186]. For more details of the contributions of experiments in non-human primates, consult Table 4.

Due to rapid advances in technology, in vitro studies are widely used. Furthermore, for ethical reasons, it is not currently possible to test on totally healthy animals without a highly relevant justification [187].

Other benefits of working with animal models include the ability to manipulate the genetic variety, ensuring that the phenotype is representative of the lesion, a small but significant statistical sample, and the ability to control environmental conditions [190]. Ethical problems derived from the use of control groups (with no treatment) and informed consent are eliminated. However, the complexity of mechanisms and organic interactions of perinatal asphyxia, as well as the presence of multiple comorbidities, particularly those of neurological development, cause animal studies to vary in terms of species, injury methods, metabolic approach, and biospecific sample, among other factors, as can be seen in the following table of revised animal models [26,30,61,70,71,159,162,165,166,167,169,170,171,172,173,174,178,179,180,181,182,186,188,189,191,192,193,194,195,196,197].

## 13. Conclusions

Perinatal asphyxia remains a significant cause of morbidity and neurological mortality in newborns. Hypoxic–ischemic encephalopathy is the clinical manifestation of generalized disordered neurologic function due to hypoxia. The pathophysiology of asphyxia is extraordinarily complex and related to several gestational, obstetric, and fetal risk factors. Mechanisms are closely related, and all contribute, to a greater or lesser extent, to primary and secondary neuronal and brain injury. Many clinical and biochemical markers have been used to evaluate intrapartum injury, but controversies remain. Recent studies have identified several biomarkers that may improve the identification of neonatal neurologic damage in the period shortly after birth. The most reliable approach to identify and evaluate the seriousness, timing, and pattern of these kinds of insults may consist in measuring an array of inflammatory and neuronal biomarkers at a precise point of care at various intervals. Diverse strategies of neuroprotection have been performed with different therapeutic targets to limit the neuronal damage associated with perinatal hypoxia and to improve the functional prognosis of newborns. Current evidence indicates that therapeutic hypothermia should start as soon as indicated to obtain the best neuroprotective results. Research in animal models has significantly contributed and provided the evidence base for the therapeutic treatment of newborns with NHIE. However, the complexity of mechanisms and interactions of perinatal asphyxia cause animal studies to vary in terms of species, injury methods, metabolic approach, and biospecific sample, among other factors. Various experimental approaches to treat neonatal HIE have advanced to the stage of clinical applications in recent years, but more optimal animal models, additional support/sponsorship from industry, and greater utilization of juvenile toxicology are all urgently required. These aspects could be complemented by dose-ranging studies based on pharmacokinetic–pharmacodynamic modeling and carefully programmed clinical trials that do not expose subjects to harmful medications or result in abandonment of potential treatments. Further investigation should focus on add-on treatment modalities or interventions to further improve outcomes.

## Figures and Tables

**Table 1:** Relevant findings of studies related to perinatal asphyxia in sheep.

Species/ Models	Objective	Contribution	Authors
**Ovine (fetuses)**	Effects of dexamethasone on brain injury due to asphyxia using one dose and a clinically relevant form of administration (12 mg of maternal IM)	It highlights the possible adverse neural effects of glucocorticoid treatment before perinatal asphyxia	[166]
**Ovine (lambs)**	1. To determine the effects on the survival and the behavior of the lamb of a brief asphyxial attack induced by occlusion of the umbilical cord at 132 days of gestation 2. To report the type and distribution of brain injury present in the newborn after an asphyxia event at 132 days of gestation.	It was shown that brief fetal asphyxia in the uterus in late pregnancy, increase the probability of premature delivery, and the lambs have significant behavioral deficits after birth that appear to arise from the underlying neuropathology caused by asphyxia, and not from premature delivery *per se*. They identified specific areas of the brain vulnerable to hypoxic damage in late pregnancy.	[167]
**Ovine (fetuses)**	To determine the changes in the regional blood flow of the fetal sheep during severe asphyxia, and with neurological damage (presence of seizures)	The pattern of redistribution of the blood flow of the ovine fetus exposed to severe asphyxia is comparable to the response of the mild asphyxia, except that a significant increase in total cerebral blood flow does not occur, a relevant finding in the likely association with the development of long-term neurological damage	[165]
**Ovine** **(fetuses)**	To evaluate the consequences of acute hypoxia on arterial and central venous pressures, carotid and femoral blood flows and HR in intact and carotid denervated fetal sheep.	The initial cardiovascular responses to hypoxia in the near-term sheep fetus have a strong carotid chemoreflex component. Moreover, fetal survival during hypoxia is dependent on this chemoreflex and the release of catecholamines from the adrenal medulla.	[61]
**Ovine** **(Fetuses)**	To evaluate the role of oxidative stress in asphyxia induced perinatal brain injury in near-term fetal lambs subjected to umbilical cord occlusion	Authors suggest that the developing telencephalic white matter seems to be most vulnerable to the effects of intrauterine fetal asphyxia and that oxidative stress may be a significant contributing factor in the pathogenesis of perinatal HIE	[71]

**Table 2:** Relevant findings of studies related to perinatal asphyxia in piglets.

Species/ Models	Objective	Contribution	Authors
**Porcine** **(Piglets)**	To examine the relationship between isovolumic relaxation time constant (IVR Tau), functional heart parameters. and heart rate (HR) during normoxia and hypoxia–asphyxia (HA) in newborn piglets.	It was demonstrated that HR and IVR Tau significantly accoupled in normoxia; however, they uncoupled during hypoxia–asphyxia (HA) in a piglet model of asphyxia.	[171]
**Porcine (Piglets)**	To establish methods for free DNA evaluation from circulant cells (cfDNA) and to investigate the temporary changes of cfDNA in blood for a clinically relevant piglet model of hypoxia–reoxygenation.	First methodological study for the extraction and evaluation of cfADN using a piglet model of hypoxia–reoxygenation. cfADN could be an early indicator of the damage caused by perinatal asphyxia.	[160]
**Porcine** **(Piglets)**	To investigate whether different metabolomic profiles are produced according to the oxygen administered during resuscitation.	The results indicated that the use of 21% oxygen seems to be better for resuscitation in piglets with normocapnic hypoxia.	[172]
**Porcine (Piglets)**	To evaluate the effects of asphyxia and resuscitation with different concentrations of oxygen on plasma metabolites in newborn piglets.	Identification of a set of markers with good correlation with the duration of hypoxia. Plasma metabolites indicated an earlier recovery of mitochondrial function when 21% oxygen is used for resuscitation compared to 100% oxygen.	[173]
**Porcine (Piglets)**	To develop an hypoxic-preconditioning (PC) model of ischemic tolerance in newborn piglets that imitates relevant clinical similarities to humans with birth asphyxia and to characterize some of the molecular mechanisms implicated in PC-induced neuroprotection in rodent models.	Results confirm, for the first time, the protective efficacy of PC against hypoxic–ischemic injury in a newborn piglet model, which reiterates many pathophysiological features of asphyxiated human neonates. PC-induced protection in neonatal piglets may involve upregulation of VEGF.	[174]

**Table 3:** Relevant findings of studies related to perinatal asphyxia in rodents.

Species/ Models	Objective	Contribution	Authors
**Murine (Rats)**	To study the neuroprotector role of palmitoylethanolamide (PEA) on the hippocampus of a 30 day-old rat after perinatal asphyxia.	Treatment with PEA (10 mg/kg) during the first hour of life could attenuate the alterations induced by perinatal asphyxia in the CA1 hippocampus neurons. Hence, PEA represents a recognized protective agent for hippocampal disorders.	[26]
**Murine (Fetal Rats)**	To investigate the acute changes that occur in the sphingomyelin/ceramide pathway after sublethal fetal asphyxia injury. To identify relevant molecules for brain tolerance.	Acute and persistent prenatal and postnatal changes in the metabolism of ceramide were found in rat brain under asphyxia, leading to positive regulation of ceramide and an increase in apoptosis.	[179]
**Murine (Rats)**	To evaluate the kinetics of arginine–vasopressin (AVP)/copeptin release during asphyxia and validate the use of the current rodent model in preclinical work on asphyxia at birth	Demonstrated that the proposed rat model meets the standard acid–base criteria for the diagnosis of asphyxia at birth and identified the production of a massive wave of AVP.	[30]
**Murine (Rats)**	To assess whether the ifetime exposure to an enriched environment (EE) (18 months) could counteract the cognitive anomalies observed in middle-aged rats that suffered 19 minutes of asphyxia at birth.	Lifelong EE was able to counteract cognitive anomalies and improved the performance of spatial learning. Results support the relevance of EE across the lifespan to prevent cognitive deficits induced by perinatal asphyxia.	[180]
**Murine** **(Rats)**	To evaluate the effects of both the physiological body temperature (33 ◦C) and excessive body temperature (37 and 39 ◦C) in neonatal rats exposed to a severe anoxia and of post-anoxic chelation of iron in neonatal rats exposed to both critical anoxia and hyperthermia on stress responses of the animals at the age of 4 months.	Authors concluded that permanent post-anoxic behavioral disorders are caused by iron-dependent oxidative brain injury, which can be prevented by reducing neonatal body temperature.	[181]
**Guinea Pigs**	To determine whether sildenafil increased fetal weight and favored fetal tolerance to induced asphyxia at birth.	Low doses of sildenafil administered from day 35 to the end of pregnancy favored fetal tolerability of intrapartum-induced asphyxia. High doses of sildenafil increased fetal weight.	[182]

**Table 4:** Relevant findings of studies related to perinatal asphyxia in primates.

Species/ Models	Objective	Contribution	Authors
**Primates** **(*Macaca nemestrina*)**	To investigate the sse of metabolomic and analysis tools to detect potential biomarkers of perinatal asphyxia. To evaluate a model of asphyxia by clamping the umbilical cord and to evaluate the differences between pre- and post-asphyxia.	Through metabolomic analyses, a profile of metabolites was identified with a significant elevation in response to asphyxia at birth (succinic acid, lactate, glucose, malate, arachidonic acid, glutamate, and butanoic acid, among others).	[188]
**Primates** **(*Macaca nemestrina*)**	To evaluate the safety and efficacy of erythropoietin (EPO) plus hypothermia for the treatment of perinatal HIE in a non-human primate model. To characterize the acute and chronic consequences of perinatal asphyxia with diagnostic imaging tools to correlate brain injury and neurodevelopmental tests to evaluate early motor and cognitive outcomes.	Occlusion of the umbilical cord for between 15 and 18 minutes can induce severe asphyxia at birth. Asphyxiated neonates developed long-term physical and cognitive deficits.	[159]
**Primates (*Macaca nemestrina*)**	To establish a non-human primate model of perinatal asphyxia suitable for preclinical evaluation of neuroprotective treatment strategies in conditions resembling human neonatal emergencies and testing erythropoietin neuroprotective treatment.	The model demonstrated changes in magnetic resonance/spectroscopy images consistent with hypoxia, significant motor and behavioral anomalies, and evidence of brain gliosis and was found to be an appropriate model of moderate-to-severe perinatal hypoxic–ischemic injury	[189]
